# Factors Associated with Postoperative Pain in Endodontic Therapy

**Published:** 2014-12

**Authors:** Durre Sadaf, Muhammad Zubair Ahmad

**Affiliations:** Dental College, Qassim University, Qassim, Saudi Arabia

**Keywords:** Root canal treatment, Root canal obturation, Cold lateral condensation Technique, Postoperative pain

## Abstract

**Objective:**

To assess postoperative pain in endodontic therapy and its association with clinical factors such as gender, age, tooth type, pulpal diagnosis, and preoperative pain, length of obturation and sealer extrusion.

**Study Design:**

Cross-Sectional study.

**Place and Duration of Study:**

Dental section of the Aga khan university hospital, Karachi, Pakistan from January to December 2009.

**Methodology:**

One hundred and forty patients (140) requiring endodontic therapy for molar and premolar teeth were included in this study. Local Anesthesia (2% Lidocain with 1:80,000 Epinephrine) was administered. The tooth was isolated with rubber dam. Access cavity was prepared with the help of round carbide No. 2 bur. Canal preparation was completed using crown-down technique. Access was sealed with sterile dry cotton pallet and restored temporarily with double layer of Glass ionomer cement and Cavit. After one week patients were recalled and access was re-opened, obturation was done using cold lateral condensation technique. Ca(OH)^2^ based sealer was used. Postoperative radiographs were taken. Patients were recalled after 24 hours and postobturation pain was recorded using Visual analogue scale (VAS).Data was obtained on a structured Performa. χ^2^ test was used for statistical analysis.

**Results:**

Pain was present in 42.9% of patients. Females more frequently experienced pain (65%) than males (35%). Preoperative pain was found to be significantly associated with postoperative pain (*p* value < 0.001). Obturation length was not found to be significantly associated with postoperative pain (*p* value 1.0). Sealer extrusion was not found to be significantly associated with postoperative (*P* value 0.547).

## INTRODUCTION

Endodontic therapy or root canal treatment (RCT) is performed to manage pain and eliminate infection from teeth. Pain is a personal and subjective experience (1). Generally appropriate diagnosis and relevant treatment procedures are successful in effecting a cure (2). Postoperative pain after RCT has been reported from 1.9%-48% (3, 4). Post-treatment pain severity demonstrated a steady decrease in post operative pain prevalence over time (5). It is considered to be associated with periapical inflammatory response. It may persist from few hours to many days after endodontic therapy (3). The factors responsible for postoperative pain are unclear. Mechanical, chemical and microbial factors may be responsible for peri-radicular inflammation (6).

**Figure 1:**
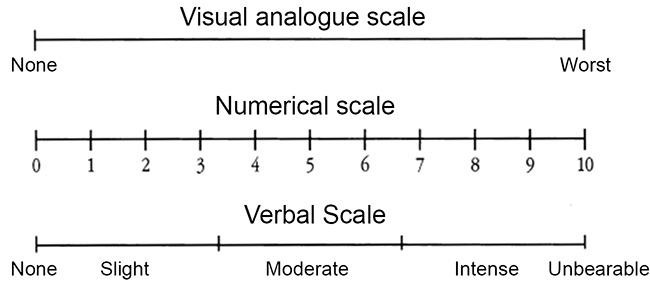
Scales for pain measurement.

Pain is a subjective experience and difficult to quantify and standardize. Pain is influenced by many factors e.g. personality, behavior, physical and psychological factors. Numerical, verbal and visual analogue scales are used in most clinical studies (7). A categorical scale composed of no pain, mild, moderate, intense and unbearable pain was used in this study (Fig. 1). The categorical scale in spite of its simplicity is considered to be a reliable and reproducible measuring tool for clinical pain trials (8).

The purpose of endodontic therapy is to perform complete debridement and obturation of root canal system and to preserve the natural dentition. Its objective is to eliminate pulpal and peri-radicular microorganisms (9). Understanding the etiology of postoperative pain after obturation may greatly help clinicians to adopt strategies to prevent such highly distressing phenomenon (10).

The purpose of this study is to assess association of clinical factors such as gender, age, tooth type, preoperative pulpal diagnosis, and preoperative pain, length of obturation and sealer extrusion with postoperative pain in endodontic therapy.

## MATERIALS AND METHODS

After obtaining permission from Ethical Review Committee of the institute One hundred and forty patients (140) visiting dental section, the Aga Khan University Hospital, Karachi, Pakistan were included in this study. Maxillary and mandibular molar and premolar teeth requiring endodontic therapy were included in this study. Third molar teeth were excluded. Patients previously on analgesics or antibiotic medications were excluded.

Preoperative pain was assessed by the application of cold (1,1,1,2 Tetrafluoroethane, TrueFlex, Ortho Technology, Inc, Tampa, Florida) and hot gutta-percha (Associated Dental Products Ltd, Kemdent Works, UK) and recorded as symptomatic or asymptomatic tooth. Pulp diagnosis was made on the basis of history and detailed clinical and radiographic examination as acute pulpitis, chronic pulpitis, pulpal necrosis and pulpal necrosis with apical periodontitis.

2% lidocain with 1:80,000 epinephrine local anesthesia (Xylestein, 3MESPE AG, Seefeld, Germany) was administered. The tooth was isolated by means of rubber dam (SafeTouch, AR Medicon Inc. Qubec, Canada). Access cavity was prepared with the help of roundcarbide No.2 bur and enlarged with Endo Z bur (Dentsply International, York, PA). Canal orifices were located and canal patency was checked with #10 K-file (Mani, Inc. Japan). Coronal and middle third was enlarged with S-1 and S-2 Protaper files (Dentsply Mallifer, Ballaigus, Switzerland). Canal orifices were enlarged with the help of Gates-Glidden burs G1-G3 (Mani, Inc. Japan). Determination ofworking length was made with Root ZX apex locator (J Morita Europe GVBH, Frankfurt, Germany) and with radiographs. Canals were prepared and shaped using crown down techniques with both manual and rotary instrumentation. During whole insturmenation RC Prep (Premier Dental Products Co. PA. USA) was used as canal lubricant and 5.25% Na HOCl was used as an irrigation solution. The canals orifices were protected with sterile dry cotton palletandthe access cavities weresealed temporarily with a double seal of Glass ionomercement (GC Corporation, Tokyo, Japan) andCavit (Meta Biomed Co. Gun Chongbuk Korea).

After one week patientswere recalled and the canals were re-opened, and obturation was performed using cold lateral condensation technique. Postoperative radiographs were taken. Radiographic length of root canal filling and sealer extrusion was recorded. Patients were recalled 24 hours after obturationand. Each patient received instructions on how to use a 10-cm visual analogue scale (VAS) to record postoperative pain (11). Immediately after each patient self recorded his/her pain, he/she was informed about the aim of the study. After that this score was transferred to a numerical value between 0 and 10 and to a verbal scale (none, mild, moderate, intense and unbearable) (Fig. 1).

Results were analyzed using SPSS 19.0. χ^2^ test was used for statistical analysis at 95% confidence interval. A p value of less than 0.05 was taken as statistically significant.

## RESULTS

**Figure 2:**
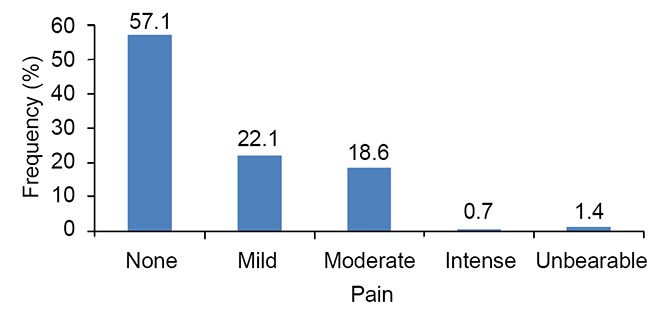
Frequency of postoperative pain.

Postoperative pain after obturation during the follow –up period of 24 hours was recorded. Pain was present in 42.9%. The pain experienced was mild, moderate, andintense in 22.1%, 18.6% and 0.7% of the casesrespectively. 1.4% of patients experienced unbearable pain (Fig. 2). Frequency and percentages of gender, tooth type, preoperative pain, and diagnosis is presented in Table 1.

**Table 1:** Frequency and distribution of clinical variables

Gender	Male n (%)	59 (42.1%)
	Female n (%)	81 (57.9%)
Type of Teeth	Mandibular Molars n (%)	70 (50%)
	Mandibular Premolars n (%)	16 (11.4%)
	Maxillary Molars n (%)	38 (27.1%)
	Maxillary Premolars n (%)	16 (11.4%)
Preoperative Pain	Asymptomatic n (%)	72 (51.4%)
	Symptomatic n (%)	68 (48.6%)
Diagnosis	Acute Pulpitis n (%)	36 (25.7%)
	Chronic Pulpitis n (%)	35 (24%)
	Pulpal Necrosis n (%)	14 (10%)
	Pulpal Necrosis with Apical Periodontitis n (%)	55 (39.3%)

Gender is not associated with postoperative painsignificantly (*p* value 0.167). More females (65%) experienced post-operative pain than males (35%). Pain was observed less frequently inmaxillary molars (28.3%) than inmandibular molars (47.6%). Association oftype of tooth withpostobtuartion pain was not found significant (*p* value 0.297) (Table 2).

**Table 2:** Association of postoperative pain with other clinical variables

Clinical Variables		Post operative pain absent	Post operative pain present	*P* Value

Gender	Males	47.5%	35%	0.167
	Females	52.5%	65%	
Type of tooth	Mandibular Molars	52.5%	47.6%	0.297
	Mandibular Premolars	7.5%	16.7%	
	Maxillary Molars	26.3%	28.3%	
	Maxillary Premolars	13.8%	8.3%	
Preoperative pain	Asymptomatic	77.5%	16.7%	<0.001
	Symptomatic	22.5%	83.3%	
Diagnosis	Acute Pulpitis	21.3%	31.7%	0.431
	Chronic Pulpitis	26.3%	23.3%	
	Pulpal necrosis	12.5%	6.7%	
	Pulpal necrosis with apical periodontitis	40%	38.3%	
Length of Obturation	within 2mm	91.3%	91.7%	1.0
	short	5%	5%	
	overextended	3.8%	3.3%	
Sealer extrusion	Present	21.3%	26.7%	0.547
	Absent	78.8%	73.3%	

Chi Square test (Fisher’s Exact test where applicable) is used at 95% confidence interval, α =5%.

83.3% preoperative symptomatic teeth presented with postoperative pain as compared to 16.7% asymptomatic teeth that presented with postoperative pain (Table 2). Preoperative pain was found to be significantly associated with postoperative pain (*p* value < 0.001).Obturation length was not significantly associated with post-operative pain (*p* value 1.00) (Table 2).

Sealer extrusion is not associated significantly with postoperative pain (*P* value 0.547).

## DISCUSSION

In this study a visual analogue scale was used to assess postoperative pain. This is a valid and reliable method which has been widely used in endodontic literature (12-14). Patients were informed the aim of the study after self recorded their pain level. In this manner, the Hawthorne effect i.e. the mere awareness of participants in an investigation can alter the way in which a person behaves, was minimized (15).

There are few studies to assess postoperative pain in endodontic therapy (16, 17). Results of this study showed that postoperative pain was absent in 57.1% but 22.1 % felt mild pain and 18.6% have moderate pain. Our results are comparable to other studies, Segura-Egea *et al* reported moderate to intense pain in 12% (16).

Greater pain has been reported in women than men (17). Reduced pain threshold is being observed in females than males (18). According to Polycarpou *et al.* female gender was considered important risk factor associated with persistent pain after endodontic therapy (13). This study also supports these reports. In this study, females more frequently experienced pain (65%) than males (35%).

Tooth type is not associated significantly with postoperative pain in this study (*p* value 0.297). Similar results are found in other studies (19, 21). Mandibular molars had greater moderate pain (50%) than maxillary molars (34.6%). These results are similar to other studies that described greater moderate pain in mandibular molars than maxillary molars. (16, 21) Results of this study disagrees with Ryan *et al* (22), who presented gender (female) and tooth type (molars) as factors that significantly influenced postoperative pain.

This study showed that teeth with acute pulpitis (34.5%) had greater moderate pain than teeth with chronic pulpitis (23.1%), pulp necrosis (11.5%) and apical periodontitis (30.8%). These results are similar to Dummer *et al*. (23) who found that 87% of patients who suffered from acute pulpitis reported severe pain.

In this study preoperative pain is associated significantly with postoperative pain (*p* value < 0.001). Teeth presented with preoperative pain more frequently experienced postoperative pain (83.3%) than previously asymptomatic teeth (16.7%). Similar results were found by Abdel Hameed *et al.* (24) which showed greater incidence of postoperative pain (15.9%)in preoperatively symptomatic teeth than in asymptomatic teeth (7.1%). Association of preoperative pain with postoperative pain has been documented in other studies as well (3, 21, 25). Sealer extrusion had not any association with postoperative pain (*p* value 0.547).

## CONCLUSION

Female gender, mandibular molar teeth and presence of preoperative symptoms are risk factors associated with postoperative pain in endodontic therapy. A significant association of presence of preoperative symptoms with postoperative pain was observed.
